# Understanding glioblastoma at the single-cell level: Recent advances and future challenges

**DOI:** 10.1371/journal.pbio.3002640

**Published:** 2024-05-30

**Authors:** Yahaya A Yabo, Dieter Henrik Heiland

**Affiliations:** 1 Translational Neurosurgery, Friedrich-Alexander University Erlangen-Nürnberg, Erlangen, Germany; 2 Microenvironment and Immunology Research Laboratory, Friedrich-Alexander University Erlangen-Nürnberg, Erlangen, Germany; 3 Department of Neurosurgery, University Hospital Erlangen, Friedrich-Alexander University Erlangen-Nürnberg, Erlangen, Germany; 4 Department of Neurosurgery, Faculty of Medicine, Medical Center University of Freiburg, Freiburg, Germany; 5 Department of Neurological Surgery, Northwestern University Feinberg School of Medicine, Chicago, Illinois, United States of America; 6 German Cancer Consortium (DKTK) partner site, Freiburg, Germany

## Abstract

Glioblastoma, the most aggressive and prevalent form of primary brain tumor, is characterized by rapid growth, diffuse infiltration, and resistance to therapies. Intrinsic heterogeneity and cellular plasticity contribute to its rapid progression under therapy; therefore, there is a need to fully understand these tumors at a single-cell level. Over the past decade, single-cell transcriptomics has enabled the molecular characterization of individual cells within glioblastomas, providing previously unattainable insights into the genetic and molecular features that drive tumorigenesis, disease progression, and therapy resistance. However, despite advances in single-cell technologies, challenges such as high costs, complex data analysis and interpretation, and difficulties in translating findings into clinical practice persist. As single-cell technologies are developed further, more insights into the cellular and molecular heterogeneity of glioblastomas are expected, which will help guide the development of personalized and effective therapies, thereby improving prognosis and quality of life for patients.

## Abstract 2

Glioblastoma, an intrinsically heterogeneous cancer, is characterized by rapid growth, diffuse infiltration, and resistance to therapies. This Essay advocates for looking at glioblastoma at a single-cell level to understand this heterogeneity, in the hope that insights gained will guide the development of personalized and effective therapies.

## Introduction

Therapy of central nervous system (CNS) tumors, which include various malignancies affecting the brain and spinal cord, poses a remarkable challenge in the field of neuro-oncology [1]. Within this group, glioblastoma, the most aggressive and prevalent form of primary brain tumor, represents an as yet incurable disease [2]. This malignancy is known for its rapid growth and diffuse infiltration, making it highly resistant to most adjuvant therapeutic strategies. Moreover, despite the strides made in surgical and medical treatment modalities, the prognosis for patients with glioblastoma remains poor, with a modest median survival time of approximately 15 months [3].

Glioblastoma is characterized by extensive inter- and intratumoral heterogeneity that was initially thought to be established by glioma stem cells (GSCs) or tumor-initiating cells. GSCs with self-renewal and differentiation capacity have traditionally been associated with tumor initiation, progression, and recurrence [4–6]. However, recent advances in the field have challenged the GSC model and instead highlighted the concept of cellular plasticity [7–11]. Glioblastoma cells exhibit a gradient of transcriptomic states and possess the ability to dynamically transit between GSC-like and differentiated states in response to microenvironmental cues and therapeutic pressure [12]. The plasticity theory therefore challenges the traditional hierarchical model of GSCs by suggesting a more adaptable and reversible tumor ecosystem [13]. This paradigm shift towards understanding the interactions between GSCs and cellular plasticity opens new avenues for targeting the elusive and dynamic nature of glioblastoma.

The complexity and heterogeneity of glioblastomas has long been an obstacle to therapeutic advances, but novel technologies are offering a new way to understand these complex malignancies [2]. Among these, single-cell and spatially resolved transcriptomics have emerged as the most promising tools. These innovative approaches allow for the molecular characterization of individual cells within complex tissues, offering a level of detail previously unattainable in cancer research. Single-cell genomic studies are providing unprecedented insights into the molecular underpinnings of tumor heterogeneity and complexity to aid the development of more precisely targeted and effective therapeutic strategies. Recent studies have revealed the cellular heterogeneity and plasticity of cancer cells, their developmental trajectories, and complex interaction within the tumor ecosystem [8,12,14–16]. By examining individual cells, researchers can discern specific genetic and molecular features driving tumorigenesis, disease progression, and therapy resistance. These technologies have shed light on the diverse cellular landscapes within glioblastomas, illuminating the presence of distinct cellular subpopulations that contribute to tumor heterogeneity and evolution. Furthermore, novel techniques and algorithms are continually being developed to enhance the quality and interpretability of single-cell genomics data [17,18].

The benefits of single-cell genomics technologies are numerous, but major challenges hinder their use in glioblastoma research [18,19]. The high cost of these technologies and complexity of data analyses and interpretation, as well as the difficulty in translating findings to improve the decision-making process in clinical practice are among the hurdles researchers in this field continue to face. Up to now, only a limited number of breakthroughs in neuro-oncology and oncology research at large have been driven by single-cell genomics, leading to a somewhat critical perspective on this technology. While the potential value and opportunities presented by single-cell genomics are significant, there is an urgent need for improvements in integrating these technologies with functional validation processes to advance the field.

As these technologies evolve and mature, they will likely offer more insights into the cellular and molecular heterogeneity of glioblastoma that are needed for modern personalized treatment strategies. The goal is that these advances will eventually translate into improved prognosis and quality of life for patients with glioblastoma. In this Essay, we therefore delve into recent advances, emerging technologies, and novel algorithms in the realm of single-cell genomics for glioblastoma. We focus on the paradigm shift from traditional single-cell transcriptomics to the use of integrated multiomics datasets in predictive modeling that will ultimately help to improve clinical decision processes. Our aim is to discuss the evolving landscape of single-cell genomics in glioblastoma research and provide a comprehensive overview, while highlighting the potential of these cutting-edge technologies to reshape our understanding and accelerate drug discovery and treatment success in glioblastoma, and CNS tumors in general.

## Understanding CNS tumors at a single-cell level

### Tumor cell heterogeneity and plasticity

Malignant brain tumors of the CNS are known for their intratumoral heterogeneity, which contributes to therapy resistance and disease recurrence [13]. Single-cell transcriptomics has emerged as a powerful tool for studying tumor heterogeneity, enabling the identification of cellular states, trajectories, and plasticity, spanning from cancer stem-like cells to differentiated tumor cells. In the early phase of the single-cell era, single-cell RNA sequencing (scRNA-seq) was used to map the heterogeneity of glioblastoma, revealing a degree of complexity and variation within these tumors that had not been fully appreciated before [14]. The previously described transcriptional phenotypes from bulk transcriptome analysis, namely, “classical,” “mesenchymal,” and “proneural,” were shown to be highly heterogeneously distributed within the tumors [20].

These investigations opened the doors for a deeper exploration of other CNS malignancies, including oligodendroglioma, where research revealed a distinct developmental hierarchy [21] that highlighted the potential for tumors to evolve and progress over time in a manner reminiscent of normal brain development. In isocitrate dehydrogenase-mutated gliomas, the influences of genetic alterations, cell lineage, and the tumor ecosystem were explored [22]. The results of this study suggest that the evolution of gliomas is not only shaped by their genetic anomalies, but also importantly stems from their cellular origins and microenvironmental interplay. Further emphasizing the role of development in glioma, an investigation into H3K27M-gliomas revealed both developmental and oncogenic processes to be important within the tumor [23]. Further research has revealed that glioblastomas mirror a normal neurodevelopmental hierarchy, suggesting that the tumor may hijack developmental pathways for its proliferation [8].

**Fig 1:**
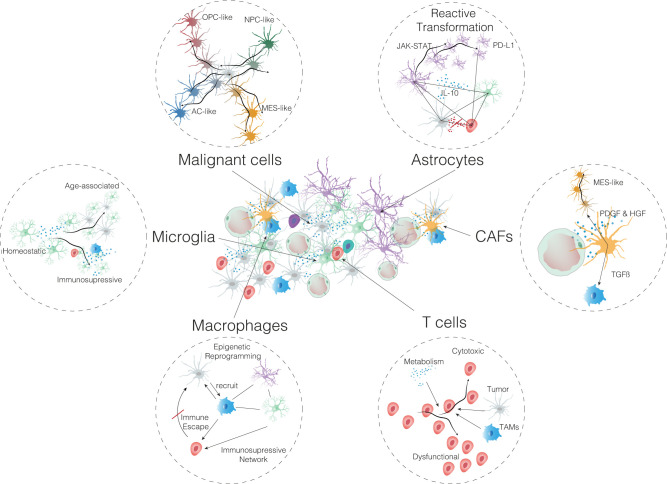
Complex interactions in the glioblastoma tumor microenvironment. Illustration of cellular differentiation and dynamic adaptation, as well as interactions in the complex microenvironment of glioblastomas. AC, astrocyte; CAF, cancer-associated fibroblast; HGF, hepatocyte growth factor; IL-10, interleukin 10; JAK, Janus kinase; MES, mesenchymal; NPC, neural progenitor cell; OPC, oligodendrocyte progenitor cell; PDGF, platelet-derived growth factor; PD-L1, programmed death-ligand 1; STAT, signal transducer and activator of transcription; TAM, tumor-associated macrophage; TGFβ, transforming growth factor β.

Investigating the disease from a spatial perspective, scRNA-seq has been used to study infiltrating neoplastic cells at the migrating front of human glioblastoma [24]. These data added to our understanding of how these tumors infiltrate into surrounding brain tissue. The most comprehensive single-cell study to date has suggested that glioblastoma cells exist in 4 distinct cellular states, namely, astrocyte-like (AC-like), mesenchymal-like (MES-like), oligodendrocyte progenitor cell-like (OPC-like), and neural progenitor cell-like (NPC-like), each of which mirrors a different developmental lineage [12]. Using mouse models, the authors show that these cellular states demonstrate a high degree of plasticity and are shaped by the tumor microenvironment (TME). The relative frequency of these cellular states in a glioblastoma tissue sample can be influenced by specific genetic amplifications and mutations, underlining the genetic complexity of the disease [12] (Fig 1). Through the technological advantage of spatially resolved transcriptomics, a recent study demonstrated that these 4 states are heterogeneously distributed across spatial niches and are linked to loco-regional inflammation and metabolic stress [16].

In addition to the investigation of primary tumors, recent research has focused on the evolution of glioblastoma and alterations during tumor progression and resistance. A single-cell atlas of glioblastoma evolution under therapy has been constructed, which revealed both cell-intrinsic and cell-extrinsic therapeutic vulnerabilities [25]. This study, along with another report [26], elegantly demonstrated the enhanced abundance of MES-like glioblastoma cells and increased inflammation within the TME. These results further elucidate the dynamic nature of glioblastoma and underscore the critical role of the TME, especially the immune system and neural stem cells, in modulating the response to therapy.

### The tumor microenvironment in glioblastoma

The TME, comprising a complex network of nontumor cells such as endothelial cells, immune cells, and astrocytes, as well as extracellular matrix components, has a crucial role in the progression of glioblastoma. It actively interacts with glioblastoma cells, influencing tumor growth, infiltration, and resistance to therapy (Fig 1). Single-cell genomics has emerged as a transformative tool in studying the TME, enabling a granular view of cellular heterogeneity. Recent studies in both tissue samples from patients with glioblastoma and in mouse models of the disease have started to unravel the composition of the glioblastoma TME [27,28].

The immune microenvironment in glioblastoma is characterized by a state of immunosuppression, often marked by the presence of tumor-associated macrophages (TAMs) and myeloid-derived suppressor cells, which promote tumor progression [27,28]. Similarly, the contribution of the vascular microenvironment is also noteworthy, as glioblastomas exhibit robust angiogenesis. Several studies have provided insights into the dynamic and complex interactions within the TME of glioblastoma, focusing specifically on the role of myeloid and other immune cells [29–31]. These studies have demonstrated that myeloid cells drive the transformation towards MES-like states in glioblastoma [31], highlighting the phenotypic plasticity of cancer cells and furthering our understanding of the complex heterogeneity that is characteristic of glioblastoma. Expanding on this, tumor cells were shown to acquire myeloid-affiliated transcriptional programs through a process known as epigenetic immunoediting to elicit immune evasion [29]. By adopting this strategy, glioblastoma can evade detection and subsequent destruction by the immune system. Furthermore, myeloid cells in glioblastoma were profiled at the single-cell level showing a remarkable degree of macrophage competition and specialization, illuminating the diversity and adaptability of myeloid cells in response to tumor evolution [28]. These findings contribute to a growing body of evidence suggesting that the TME is a dynamic and adaptive system in which macrophages, for example, might adjust their behavior based on signals from their surroundings.

Further broadening our understanding of the glioblastoma microenvironment, an in-depth investigation into the states of microglia, the resident immune cells of the brain, was performed through the integration of multiple high-dimensional techniques [30]. The findings of this study provide valuable insights into the functional diversity of microglia in both health and disease, further emphasizing the importance of immune elements in glioblastoma pathophysiology. scRNA-seq and cellular indexing of transcriptomes and epitopes sequencing (CITE-seq) profiling of tissue samples from patients with glioblastoma and from mouse models of glioma revealed significant compositional and expression differences between myeloid cells from males and females [27]. In the patient samples, the proportion of various cellular populations varied between men and women, with men having a higher proportion of bone marrow-derived macrophages (BDMs) and women having a higher number of microglia. Microglia and a subset of BDMs from men differentially expressed MHC class II genes in comparison to those from women. Further analysis confirmed a higher enrichment of tumor-supportive genes, in addition to MHC class II and costimulatory molecule PD-L1 expression, in men, while the myeloid cells from women were enriched for interferon gene expression. These results suggest that the difference in survival between men and women with glioblastoma may be linked to the identified differences in myeloid cell composition and gene expression. This indicates that sex difference is an important variable that should be properly accounted for in the analysis of transcriptomic data to rule out any potential bias that may be introduced by the sex of the patients.

A study investigated glioma-infiltrating T cells using single-cell transcriptomics and T cell receptor (TCR) sequencing, leading to the identification of CD161 as an inhibitory receptor [32]. This receptor represents a previously unrecognized aspect of the immunosuppressive TME in glioblastomas. The presence of this inhibitory receptor explains some of the challenges encountered in stimulating effective antitumor immune responses and suggests that therapeutic approaches may need to consider methods for blocking or circumventing this inhibitory signal. Another study examined the factors contributing to T cell dysfunction in the glioblastoma TME [33]. The authors demonstrated that a defined subpopulation of heme oxygenase 1-expressing myeloid cells release the cytokine interleukin 10 (IL-10), which, in turn, mediates T cell dysfunction. The precise elucidation of immune suppression mechanisms at play within the glioblastoma TME underscores the importance of understanding cellular communication and interaction within the tumor ecosystem.

Single-cell sequencing has also been instrumental to our current understanding of the complexity of neuron–glioma interactions by helping researchers to identify and describe the role of the different subpopulations involved in glioblastoma infiltration and colonization of the brain. A subpopulation of glioma cells with neuronal and neural progenitor-like cell states were found to integrate into neuronal circuits and co-opt neuronal mechanisms to fuel tumor progression and invasion in xenograft models [34]. scRNA-seq was also used to delineate glioblastoma tumors into high functionally connected (HFC) and low functionally connected (LFC) regions [35]. HFC regions had a higher expression of *THBS1*, which facilitates the formation of neuron–glioma connectivity, higher tumor growth, and lower survival, compared to tumors in LFC regions in glioblastoma xenografted mouse models. Interestingly, *THBS1* was expressed in a compensatory manner by astrocytes and myeloid cells within the TME in the LFC tumors. These findings shed further light on the earlier findings that glioma cells physically form neuron–glioma synapses to drive tumor growth [34,36].

In glioblastoma, the influence of structural cells such as fibroblasts, pericytes, and glial cells on the growth and differentiation of tumor cells and signaling towards other cells in the tumor ecosystem is of increasing interest. Recent studies have demonstrated that tumor-associated reactive astrocytes have a key role in the evolution of an immunosuppressive environment in glioblastoma. This suggests the potential for targeting these astrocytes to alleviate immunosuppression and enhance the effectiveness of immunotherapies for treatment [37]. Immunometabolic regulation also influences the TME and drives glioblastoma pathogenicity in genetically engineered glioblastoma mouse models, emphasizing the crucial role of metabolic interactions between astrocytes and glioblastoma cells during tumor progression [38]. By integrating scRNA-seq and spatially resolved transcriptomics data, the role of cancer-associated fibroblasts (CAFs) in promoting glioblastoma has been explored. The results of this study underscore the protumoral effects of CAFs, suggesting that these cells may be a viable target for therapeutic intervention [39]. An additional interesting angle is provided by a study that illustrated the association between tumor-associated hematopoietic stem and progenitor cells and glioblastoma progression [40]. Taken together, these studies highlight the importance of the various nonmalignant cells in the TME and the need for investigating the intricate interplay between glioblastoma cells and various immune components within the TME, and their role in influencing disease progression and treatment response.

### The spatial architecture of the glioblastoma ecosystem

Single-cell genomics data, without spatial context, are limited due to their lack of information regarding the natural embedding of cells within the complex tumor ecosystem. Spatially resolved transcriptomics data, on the other hand, are either restricted in resolution (array-based methods) or biased by a predefined gene panel (in situ sequencing). Both limitations make it currently inevitable to integrate both technologies to infer the spatial architecture of glioblastomas. Most recent studies have used array-based spatial transcriptomics or multiomics to investigate recurrent regional expression patterns across samples from patients with glioblastoma. All studies confirmed that the tumor landscape is shaped by either “reactive” niches, in which the cancer cells respond to inflammatory signals or metabolic stress, or “nonreactive” niches, which mainly contain tumor cells in developmental stages such as NPC-like or OPC-like cells that more frequently interact with the neuronal environment of the brain [16,41,42]. Each of these distinct niches consists of a defined cellular neighborhood, which can be used to predict clinical behavior [43] or response to therapy [44]. A recent spatial transcriptomics study profiled the TCR repertoires of patients with glioblastoma to further our understanding of the limited presence of T cells in glioblastomas [45]. By adding histopathological characterization and signatures of tumor regions from bulk RNA-seq profiling of glioblastoma, the authors found that T cell functional diversity was associated with different tumor niches. The addition of metabolomic information further helped in segregating different T cell phenotypes. This study demonstrates that the current resolution of spatial transcriptomics can be augmented with scRNA-seq, bulk transcriptomics, metabolomics, or imaging data to deconvolute individual cells and identify their interacting partners within tumor niches.

### Experimental models of glioblastoma

The quest to find an ideal experimental model that will advance our understanding of glioblastoma has been gradual, with incremental benefits over the years. Human glioblastoma-derived cell lines are the most popular because of their low cost, accessibility, and ease of handling; in comparison to patient material, they can be easily genetically manipulated [46]. However, these cell lines evolve and develop genetic alterations that distance them from the original patient tumor. Three-dimensional stem-like cultures are better able to recapitulate human glioblastomas and are being used to study inter- and intratumoral heterogeneity and cellular plasticity [7]. Single-cell transcriptomics analysis of glioblastoma 2D cell culture systems and organoid models have demonstrated that inter- and intrapatient heterogeneity [47] is partly maintained in these models. However, the lack of the TME forces the model system towards MES-like cell states [48]. A novel ex vivo model approach, namely, “patient-derived explants,” preserves cellular heterogeneity compared to the original tumor tissue [48]. Further characterization of these glioblastoma organoid models using scRNA-seq analysis revealed the presence of some TME cell types, as well as tumor cells with features of the different cellular states previously identified in glioblastoma [47,49].

Human neocortical slices offer another alternative for ex vivo model systems. In this model, human glioblastoma cells are inoculated into human cortical brain slices [50,51]. Unlike organoids, this approach simulates tumor infiltration into healthy brain tissue. Evidence from single-cell sequencing analysis shows that tumor cells can adapt to the host environment and that their behavior is influenced by external factors such as patient age [16]. For ex vivo models, it is imperative to note that they cannot simulate the effects of therapies based on overall survival or interactions with the blood-derived cell compartment. This limitation underscores the importance of animal models, primarily rodents, as a pivotal system for glioblastoma research.

Single-cell profiling of patient-derived orthotopic xenograft models revealed that these models recapitulate the physiologically relevant glioblastoma ecosystem, with an intact blood–brain barrier and varying tumor niches within the TME [52,53]. However, these models are immunodeficient, missing the T cell component of the TME. To address this, genetically engineered mouse models have been developed [54]. These models provide insights into the relationship between genetic drivers and transcriptional plasticity, as well as interactions with various bone-derived and brain-resident cell compartments. Through scRNA-seq analysis, the impact of genetic drivers (*PDGFB*-driven or *NF1*-silenced) on glioblastoma development in genetically engineered mice has been explored; some of the results obtained so far demonstrated that the turnover of monocytes and neutrophils within the TME was dependent on the genotype of the mice [55]. Collectively, the treatment of these experimental models with different therapeutic regimens followed by the characterization of the tumor cells using single-cell transcriptomics has so far advanced our understanding of various mechanisms of tumor cell proliferation, invasion, plasticity, and treatment resistance [44,47].

## Advances in single-cell technologies in glioblastoma research

**Fig 2:**
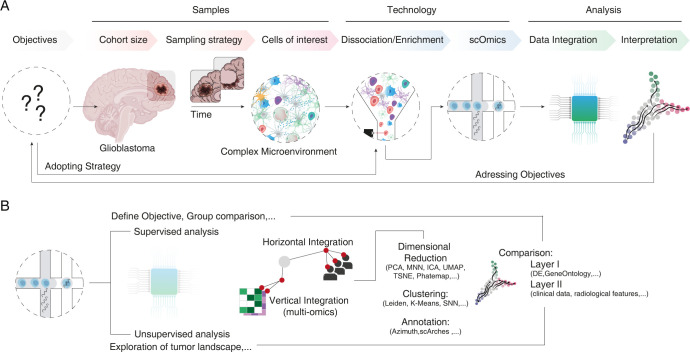
Overview of the conceptual design of single-cell glioblastoma studies. **(A)** Illustration of the steps in study design of single-cell experiments. (**B**) Illustration of the steps in single-cell analysis. DE, differential expression; ICA, independent component analysis; MNN, mutual nearest neighbor; PCA, principal component analysis; sc, single-cell; SNN, shared nearest neighbor; TSNE, t-distributed stochastic neighbor embedding; UMAP, uniform manifold approximation and projection.

As discussed in the previous section, single-cell transcriptomics has been instrumental to recent advances in the understanding of glioblastoma biology. Fig 2 provides an overview of the design of a typical single-cell transcriptomic study in glioblastoma.

### State-of-the-art single-cell genomics

In recent years, there have been significant leaps forward in single-cell genomics technologies [56]. These advances have opened up new possibilities for exploring biological systems with scalable resolution. Today, it is possible to analyze thousands of individual cells simultaneously, which has been instrumental in uncovering the complex cellular heterogeneity of glioblastoma and for revealing novel biological insights. At the forefront of these technological developments are droplet-based microfluidics and nanowell-based platforms [57]. The Chromium System from 10X Genomics is an example of an enhanced droplet-based microfluidics platform that has significantly scaled-up the throughput of single-cell transcriptomics experiments [58]. This system facilitates the encapsulation of individual cells in microdroplets, each of which is embedded with a uniquely barcoded bead, allowing simultaneous transcriptomic analysis of thousands of cells. However, single-cell transcriptomics is prone to bias due to the dissociation protocol or gating strategy used, which can lead to the loss of specific cell types, including neurons [59]. To address this, recent research has turned to single-nucleus RNA sequencing (snRNA-seq), which offers a more comprehensive view of cellular distribution in glioblastoma [25]. Although snRNA-seq has been mainly used to profile frozen specimens [60], an additional benefit is its compatibility with paraffin-embedded tissue, which grants researchers access to expansive biobanks. This access, in turn, enables the study of a more substantial number of samples, promising a broader understanding based on larger cohort sizes in future research [61].

### Multiomics platforms

The landscape of sequencing technologies has seen a rapid evolution, with enhancements in scRNA-seq techniques and the advent of multiomics methodologies that enable simultaneous examination of the transcriptome, epigenome, and proteome within individual cells. These technological advances have unveiled a more detailed and holistic understanding of the cellular states and molecular activities in conditions such as glioblastoma. A recent study combining these multiomics methodologies and cellular tracing provided a more comprehensive insight into the evolution of glioblastoma under therapy [25]. However, the use of these stand-alone methodologies is cumbersome due to the limited amount of tumor specimen that gets to the bench. Recently, commercially available solutions for multiomics analyses have been gaining popularity. Platforms such as 10X Genomics’ multiomics systems, which integrate assay for transposase-accessible chromatin with sequencing (ATAC-seq), CITE-seq, and RNA-seq, provide the research community with a comprehensive toolkit to explore single-cell genomics in an in-depth and integrated manner [62]. The integration of epigenetic layers such as via ATAC-seq [25] or DNA methylation analysis [15,63] has revealed dysregulated epigenetic mechanisms underlying gliomagenesis. However, the multiomics landscape extends beyond these platforms, with a range of technologies available that are tailored to address diverse scientific questions. Tools for multiomics identification of TCRs and B cell receptors [45], mitochondrial DNA (via PHAGE-ATAC) [64], and single nucleotide variations or copy number variations (using G&T-seq or scGET-seq) are now readily available [65,66]. Additional methods are designed to examine methylation patterns (via scMT-seq and scNOMe-seq) [67,68], study dynamic RNA turnover (through scEU-seq) [69], or carry out perturbation experiments (via Perturb-seq, CROP-seq, or sci-Plex) [70–72]. Furthermore, the sphere of multiomics applications has been expanding into the realm of spatial resolution. This progress in the field enables researchers to investigate not only the multiomics signature of individual cells but also their spatial organization and interactions within the tissue environment.

## Challenges in implementing single-cell transcriptomics for glioblastoma research

### Study design

As in any biological or clinical research, proper study design is fundamental for obtaining reliable and valuable results in glioblastoma research [73] (Fig 2). The predetermined objectives of the study guide the definition of the cohort, sample sizes, and sampling strategies [74]. This is particularly crucial in glioblastoma research, where precise neurosurgical sampling (including MRI-guided neuronavigation and validation of tumor content in the sample) is necessary to minimize batch effects and facilitate comparison across patients. Given the diverse spatial architecture of brain tumors, the cellular composition of the sample can vary greatly depending on the sampling region and could affect the outcome of the single-cell study. Following sample acquisition, careful consideration must be given to strategies for sample dissociation, which will depend on the chosen technology (e.g., snRNA-seq, scRNA-seq, or multiomics) [75]. After dissociation, cells are typically enriched or sorted to remove dead cells, quantify the cells, and enhance the presence of specific cell types [51]. As most single-cell technologies may not sufficiently capture all cell types, strategies must be adapted to the scientific objectives initially defined, such as using barcoding techniques to track and capture rare and transient cell populations [76]. The final stage of study design involves selecting the most appropriate method (scRNA-seq or snRNA-seq; transcriptomics, epigenetics, or proteomics) that aligns with the set objectives. Predefining the steps of the analysis can also help to avoid unjustified data dredging or “fishing for significance.”

In glioblastoma, estimating the sample size for single-cell studies is not straightforward and largely depends on the objectives, effector size, and complexity of the study [77]. Unlike in bulk sequencing studies, where power calculations are commonly employed, single-cell studies have additional layers of complexity including cellular heterogeneity and technical noise [78]. The number of cells to sequence and the sequencing depth required per sample is a critical decision that balances cost with the need to capture sufficient biological variation and rare cell types in glioblastoma. Generally, pilot studies or existing literature can inform the expected distribution of cell types and variability, aiding in sample size calculation. Larger sample sizes can increase the chance of capturing rare cell types and reduce the influence of dropout events (i.e., failure to detect an expressed gene). For studies comparing conditions or groups of patients, considerations should include not only the number of cells sequenced per condition but also the number of biological replicates (e.g., individual patients or time points). Adequate biological replicates are key to generalizing findings beyond individual-specific effect [79]. Researchers should also consider the downstream computational analyses, as some methods may require more cells or greater sequencing depth. Despite these complexities, recent methodological advances are beginning to provide more concrete guidelines for glioblastoma sample size estimation in the context of specific experimental designs and analytical goals [73].

### Computational biology

With the rising accessibility of single-cell technologies, the major hurdles to progress are shifting towards computational biology. Initial single-cell transcriptome data, containing only a limited number of cells, relied on computational tools developed for bulk RNA-seq analysis. However, as the technology advanced, traditional computational approaches proved insufficient for handling high cell numbers, multiple batch factors, and complex data integration [80]. Therefore, many fields, particularly single-cell genomics, are increasingly adopting artificial intelligence (AI)-based solutions, including deep learning (DL) technologies [81].

In the routine downstream analysis of single-cell transcriptomic studies, quality control and appropriate data filtering processes are followed by data integration, which can be horizontal or vertical. This step is further supplemented by dimensionality reduction and clustering [73,79,82,83]. Identification of cell types and/or states is then performed using defined markers or reference datasets [84–86]. After these routine analyses, there is an opportunity for data exploration and profiling of the single-cell landscape, or to perform a tailored analysis to answer predefined questions. Aspects such as experimental parameters and cohort size must be determined in advance to ensure the quality and relevance of the analysis.

The field is progressing toward the use of AI-based algorithms. A clear advantage of these algorithms is their ability to efficiently integrate and interpret large datasets. Notably, the application of transfer learning strategies (scArches or scJoint) [86,87], transformer architectures (scGPT [88] or scBERT [89]), and cycle generative adversarial networks (GANs; such as scGAN [90]) enable impressive integration of datasets from diverse platforms or diseases. Still, comprehensive benchmarking of these algorithms has remained elusive. A drawback of all DL solutions is the lack of “explainable AI,” especially in highly complex DL architectures. Further benchmark studies are necessary [91] to broaden the acceptance of DL applications in single-cell genomic analysis.

### Unraveling complex signaling in glioblastoma

Despite advances in spatial and single-cell technologies, interrogation of direct cell–cell communication has remained challenging, both experimentally and computationally. As spatial transcriptomics moves closer to single-cell resolution, it will allow the investigation of cellular signaling in different glioblastoma niches in situ. Similarly, scRNA-seq methodologies that will allow the capture and sequencing of doublets could reveal cells in direct communication [33]. Although many tools have been developed to identify cell–cell communication from scRNA-seq data, they only infer ligand–receptor interactions based on the expression of known genes that translate into ligands and receptors from existing databases. This implies that novel ligand–receptor interactions, particularly in pathologic situations such as glioblastoma, may be missed or excluded. A computational modality that integrates spatial location data at single-cell resolution with protein expression data in situ will provide more insights on cellular signaling in glioblastoma.

## Future directions for single-cell genomic studies

### From descriptive to predictive analysis

**Fig 3:**
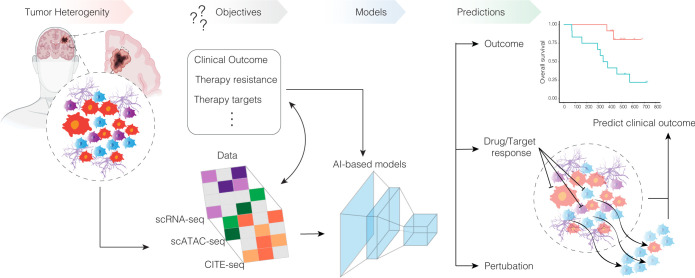
Future challenges in single-cell profiling of glioblastoma. The objective of modern multiomic single-cell analysis is to build predictive models to predict outcome and optimize tumor diagnostics; model genetic or environmental perturbations; or predict the response to drug treatments or screen for target therapies. This illustration provides an overview of the possibilities in AI-based modeling of cellular responses and clinical data integration. AI, artificial intelligence; CITE-seq, cellular indexing of transcriptomes and epitopes sequencing; scATAC-seq, single-cell assay for transposase-accessible chromatin with sequencing; scRNA-seq, single-cell RNA sequencing.

While single-cell transcriptomics has traditionally been used for descriptive analysis in glioblastoma research, the future lies in shifting toward predictive models. Rather than just describing the heterogeneity and dynamics of cellular populations, the goal is to predict cellular behaviors, responses to perturbations, or disease progression based on the single-cell data (Fig 3). Currently, there is a consensus in the field on the need to integrate single-cell transcriptomic measurements with other multiomics recordings from individual cells, such as proteomics, epigenetics, and spatial information, using machine learning (ML) algorithms to fully understand cellular patterns and predict cellular transitions in the complex biological system of glioblastoma [92,93].

The development of novel computational models and tools, possibly leveraging ML and AI, is required to comprehensively integrate single-cell data with other data types and accurately predict future cellular states or clinical parameters from a given patient sample. Early efforts to predict cellular types/states and behavior using scRNA-seq data started with stand-alone tools such as SCENIC [94], which integrates several algorithms that allow the quantification of transcription factor activity in individual cells. The further development of SCENIC+ enabled scATAC-seq and scRNA-seq data to be combined to predict genomic enhancers upstream of transcription factors and link these enhancers to target genes. This multiomics approach improves gene regulatory network (GRN) inference and increases its applicability to studying dynamic cell-state changes by predicting transcription factor perturbation effects and GRN velocity.

Another algorithm based on GRNs, CellOracle, integrates several algorithms including ML to provide a better understanding of cellular dynamics by in silico perturbation modeling. These predictive models were used to predict the impact of IL-10 signaling in the TME of glioblastoma, demonstrating a stabilization of cytotoxic T cells after IL-10 signaling inhibition [33]. To look beyond cell fate predictions along their regular trajectories, Compositional Perturbation Autoencoder, a deep generative model trained on large datasets from high-throughput single-cell perturbation experiments, was developed to predict how cells respond to perturbations [95]. This model can predict the effects of different drug dosages given at varying time points in various cell types and decode normal changes in gene expression profiles [95], as well as in multiomics datasets [96]. The rapid advancement and optimization of such computational pipelines for single-cell perturbation has the potential to accelerate drug development with a higher specificity.

Following the success of large language models, scGPT has been developed to enable multiomics and multibatch data integration. It facilitates cell type annotation, prediction of gene perturbation, and GRN inference and is based on the construction of a foundation model pretrained using over 33 million cells and a transfer learning approach [88]. Taking advantage of such advances in multiomics measurements, computational power, and methodologies such as AI, ML, and DL to decrypt the integrated multidimensional single-cell data and accurately predict the transition of cells and possible clinical outcome(s) of such transitions opens an exciting prospect for the use of single-cell technologies to elucidate the complex networks that allow glioblastoma to evade treatment. These tools and computational power can also be harnessed to speed up tumor diagnoses intraoperatively with unprecedented speed and resolution [97].

### Translation to the clinic

As single-cell genomics technologies continue to progress, their translation into clinical neuro-oncology is becoming a vital but challenging goal. There is a need to refine these techniques not only for the analysis of patient samples but also for predicting treatment and disease-related outcomes in patients with glioblastoma. For example, inference and prediction tools can aid personalized medicine by accurately predicting the effects of drug treatments or genetic modifications at the single-cell level. scRNA-seq analysis has been used to stratify patients with glioblastoma on the basis of the dominant gene expression profile in their tumors [12,20]. However, the presence of intratumoral heterogeneity implies that different tumor regions may respond differently to treatment. In addition, differences in the spatial location of tumor cells may also indicate varying clonal architecture and tumor invasion patterns that could lead to spatially distinct resistance mechanisms, as hypothesized by the spatial glioblastoma cell atlas [16].

Treatment resistance and recurrence is a major bottleneck in the clinical management of glioblastoma. Integrated single-cell transcriptomics will help in monitoring treatment responses in various tumor regions and in identifying potential resistance mechanisms at an early stage, enabling clinicians to optimize treatment protocols and monitor therapeutic effectiveness. Early detection of treatment-resistant clones or subpopulations within a tumor could help in eliminating residual disease and prevent recurrence [25]. The presence of certain TME subpopulations of cells and their interactions with glioblastoma cells dictates the transition to a specific cellular state [31]. Thus, it is important to delineate and understand critical TME factors, such as immune cell infiltrates and structural components, and how they communicate and influence tumor evolution and plasticity during therapy. When different single-cell transcriptomics modalities are integrated, with the help of advanced computational methods, they can be used to identify specific cell-to-cell interactions and signaling pathways. In addition, simulating and predicting cellular dynamics and appropriate therapeutic strategies to arrest cellular transitions during tumor evolution will aid in the understanding of glioblastoma pathogenesis and guide the development of targeted therapies.

## Conclusions

Single-cell transcriptomics has revolutionized our understanding of glioblastoma by providing an unprecedented level of detail about its molecular complexity and cellular heterogeneity. Recent advances in technologies and algorithms have further propelled this field forward, enabling the elucidation of previously unclear complex phenomena, such as dynamic tumor cell plasticity and the characterization of TME components, as well as disentangling the complex interactions between tumor and nontumor cells underpinning glioblastoma progression, therapy resistance, and recurrence. However, challenges remain, including the need for improved sensitivity and accuracy, better integration of multiomics data, and the translation of research findings into clinical applications. The high cost of single-cell technologies may also hinder its integration into routine clinical practice. Addressing these challenges and continuing to explore the intricacies of glioblastoma biology should pave the way for more effective therapies and improved patient outcomes.

In the future, glioblastoma research should focus on overcoming these hurdles through the integration of single-cell data with other omics and the development of novel computational tools that leverage advances in AI and ML. The full potential of single-cell transcriptomics will only be realized by transiting from descriptive analyses of glioblastoma biology to models that can predict cellular behaviors, disease progression, and treatment responses. As we move forward, the promise of single-cell genomics in glioblastoma lies in its potential to accelerate tumor diagnosis [97], drug discovery, and personalized treatment strategies for patients.

## Abbreviations

AC: astrocyte
AI: artificial intelligence
ATAC-seq: assay for transposase-accessible chromatin with sequencing
BDM: bone marrow-derived macrophage
CAF: cancer-associated fibroblast
CITE-seq: cellular indexing of transcriptomes and epitopes sequencing
CNS: central nervous system
DL: deep learning
GAN: generative adversarial network
GRN: gene regulatory network
GSC: glioma stem cell
HFC: high functionally connected
IL-10: interleukin 10
LFC: low functionally connected
MES: mesenchymal
ML: machine learning
NPC: neural progenitor cell
OPC: oligodendrocyte progenitor cell
scRNA-seq: single-cell RNA sequencing
snRNA-seq: single-nucleus RNA sequencing
TAM: tumor-associated macrophage
TCR: T cell receptor
TME: tumor microenvironment
